# Research Progress on Membrane Separation Technology for Oily Wastewater Treatment

**DOI:** 10.3390/toxics12110794

**Published:** 2024-10-31

**Authors:** Yichang Wang, Yu Zhang, Liang Liang, Feng Tu, Zhongjian Li, Xianjin Tang, Li Dai, Lingli Li

**Affiliations:** 1 State Grid Zhejiang Electric Power Co., Ltd. Construction Branch, Hangzhou 310008, China; wang_yichang_ee@163.com (Y.W.);; 2 Institute of Soil and Water Resources and Environment Science, College of Environment and Resource Sciences, Zhejiang University, Hangzhou 310012, China; 22214116@zju.edu.cn (Y.Z.); xianjin@zju.edu.cn (X.T.); 3 Institute of Industrial Ecology and Environment, College of Chemical and Biological Engineering, Zhejiang University, Hangzhou 310012, China; zdlizj@zju.edu.cn

**Keywords:** oily wastewater, membrane separation, dispersed oil, emulsified oil, dissolved oil, membrane bioreactor

## Abstract

This paper presents the research progress and future prospects of membrane separation technology for treating oily wastewater. It discusses various treatment methods tailored to different sources and characteristics of oily wastewater, summarizing the features of different membrane separation technologies and the latest advancements in their application. The paper concludes by emphasizing the need for future research to focus on developing environmentally friendly and efficient coupled membrane treatment technologies, optimizing membrane material design and enhancing the environmental benefits of oily wastewater treatment.

## 1. Introduction

Oily wastewater is a significant pollutant from various industries, including oil and gas extraction, food and beverage processing, and engineering construction [1,2]. During oil and gas extraction, the extracted fluid typically contains a water-to-crude oil ratio of 3:1, producing large quantities of oily wastewater [3]. In 2020, the global discharge of oily wastewater will reach 54 billion m^3^ [4]. The food, beverage, and dairy industries also contribute to substantial oily wastewater production due to the critical role of water in most products. Unlike petrochemical waste oil and marine oil spills, oily wastewater from the food industry contains a high proportion of floating and dispersed oil (>80%), which is highly prone to spreading, leading to significant environmental impacts [5]. Furthermore, various industrial processes and equipment maintenance activities generate substantial amounts of oily wastewater. The untreated discharge of oily wastewater can cause severe water and soil pollution, disrupt aquatic ecosystems, harm plant and animal health, and potentially impact human health through the food chain. Due to the complex composition of oily wastewater from different industries, tailored treatment solutions are often required for specific situations [6]. Oily wastewater can be classified into three types based on the size of oil droplets: floating, dispersed, and emulsified. Floating oil, which accounts for 70–80% of the total oil content, consists of larger oil particles (>150 μm) that are relatively easy to remove. Dispersed oil, with particle sizes between 25 and 150 μm, typically transitions to floating oil after settling. Emulsified oil, with particles smaller than 20 μm, forms an emulsion in the wastewater, stabilized by surfactants, making it difficult to separate [7]. Oily wastewater is characterized by high levels of biochemical oxygen demand (BOD), total suspended solids (TSS), chemical oxygen demand (COD), ammonia, sulfides, total organic carbon (TOC), and total petroleum hydrocarbons (TPH), with concentrations varying depending on different operations and products. Additionally, oily wastewater contains numerous toxic compounds, including volatile organic compounds (VOCs), polycyclic aromatic hydrocarbons (PAHs), phenols, and heavy metals, necessitating selecting the most appropriate treatment methods.

Common methods for treating oily wastewater include physical, chemical, and biological approaches. Physical methods are primarily used to treat wastewater containing floating and dispersed oil, employing techniques such as air flotation, sedimentation, adsorption filtration, centrifugal separation, coalescence, and membrane separation. The advancement of physical methods largely hinges on developing new separation materials. Recent innovations, including hydrogels, polyvinyl alcohol sponges, and carbon nanomaterials, have led to significant technological breakthroughs in oily wastewater treatment due to their high stability, porosity, and strong oil–water separation capabilities [8,9,10,11]. Chemical methods typically applied to wastewater containing highly dispersed and emulsified oil include flocculation, coagulation, acidification destabilization, chemical demulsification, electrochemical methods, and advanced oxidation processes. While effective, these methods can sometimes lead to secondary pollution. Among them, advanced oxidation processes have garnered considerable attention due to their ability to efficiently generate reactive oxygen species and degrade organic pollutants [12,13,14,15]. Biological treatment methods utilize microbial metabolism to convert dissolved or colloidal organic pollutants in water into harmless substances. The most mature and widely used processes include the activated sludge method and biofiltration [16]. However, the high construction costs associated with these methods often limit their large-scale adoption [17,18,19]. Among the physical methods, membrane separation technology has recently attracted increasing attention and application due to its high separation efficiency and operational simplicity.

Membrane separation is a green physical process that typically does not require adding chemical agents. It offers several advantages, including a modular design, a small footprint, and a high level of intelligent control [20,21,22]. Membrane technology can effectively handle various types and concentration ranges of oily wastewater by selecting separation membranes with appropriate pore sizes based on the size of the oil droplets. The concentrated oil obtained through membrane separation can be further processed into fuel or chemical raw materials, enabling resource recovery and utilization. Additionally, membrane separation technology is highly effective in treating wastewater with complex compositions [23]. It efficiently removes suspended and emulsified oils and significantly reduces the content of organic pollutants and toxic compounds in the wastewater.

Many countries have stringent discharge standards for oil and grease content in wastewater. For example, the U.S. Environmental Protection Agency (EPA) sets limits on the maximum concentration of oil and grease in industrial effluents, typically ranging from 10 to 15 mg/L depending on the type of discharge. Membrane technologies, such as ultrafiltration (UF) and reverse osmosis (RO), effectively reduce oil concentrations to below these regulatory limits, ensuring that industries meet legal requirements [24]. On offshore oil platforms, ceramic membranes are used to treat produced water containing oil and other contaminants. Membrane filtration systems, including ceramic and polymer membranes, are efficient for separating oil from water, even in emulsified states. This technology enables platforms to meet stringent environmental standards for water discharge [25]. In the automotive industry, membrane separation has been applied to treat oily wastewater generated from metal surface treatments and washing processes. Nanofiber membranes, for example, are used for their high efficiency in separating oil from water, achieving more than 90% separation efficiency. This water is often reused in the plant, contributing to sustainability efforts [26].

For oily wastewater treatment, Zoubeik et al. [27] reviewed recent advances in both polymer and ceramic membrane technologies. The review covered membrane modification strategies aimed at reducing membrane fouling and contamination and approaches for optimizing permeate flux to improve overall performance in wastewater treatment applications. Continuous research and development in membrane technology, along with the application of new materials, have further enhanced treatment efficiency, extended membrane lifespan, and reduced operational costs. Consequently, membrane technology is widely recognized as an efficient and environmentally friendly solution for treating oily wastewater.

## 2. Membrane Separation Technology for Oily Wastewater Treatment: Principles, Types, Performance, and Operational Modes

### 2.1. Principles of Membrane Technology for Oily Wastewater Treatment

The principle of membrane separation technology for oily wastewater treatment primarily relies on pressure-driven and crossflow filtration operations. In this process, pressure is applied to one side of the membrane, forcing the wastewater components to pass through the membrane’s selective pore sizes. The membrane’s pore size acts as a selective barrier, allowing smaller pollutants and water molecules to pass through while blocking and retaining larger oil particles and other suspended solids on the feed side of the membrane. In addition, membrane separation technology for oily wastewater treatment relies heavily on the selective wettability of the membrane. This selective wettability ensures that oil droplets do not penetrate the membrane’s pores by utilizing specific properties, such as hydrophilicity and underwater oleophobicity. These properties allow the membrane to selectively allow water to pass through while repelling oil, enhancing the efficiency of the separation process [28,29]. This selective filtration enables membrane separation technology to effectively separate oil from water, with particularly notable effectiveness in treating wastewater containing emulsified oil. Emulsified oil consists of very small oil droplets, which are challenging to remove using traditional gravity separation methods. However, membrane separation technology can efficiently capture these tiny droplets, resulting in a more thorough and effective separation [30].

### 2.2. Membrane Types

In membrane separation technology, membrane materials can be classified based on pore size into microfiltration membranes, ultrafiltration membranes, and others. Based on the material, they can be categorized into organic membranes, inorganic membranes, and composite membranes.

#### 2.2.1. Classification by Membrane Pore Size

Microfiltration membranes are widely employed for separating oily wastewater with particle sizes larger than 0.1 μm, including those from industrial, catering, and petrochemical sources. These wastewaters often contain large amounts of oils and suspended particulates, which, if discharged untreated, can lead to severe environmental pollution. The transmembrane pressure difference for microfiltration membranes typically ranges from 0.01 to 0.3 MPa, enabling effective separation at relatively low pressures. Depending on the nature of the wastewater and the material of the microfiltration membrane, the membrane flux can reach 50–200 L per square meter per hour. Microfiltration membranes are commonly made from polypropylene, ceramics, and metals, offering homogeneous porous structures with excellent filtration performance. These membranes effectively remove most oil particles and suspended solids. Compared to ultrafiltration, nanofiltration, and reverse osmosis, microfiltration membranes offer significant advantages in treating oily wastewater. Their higher membrane flux allows for treating larger volumes of wastewater, enhancing treatment efficiency and reducing costs. Consequently, microfiltration membranes are widely regarded as an economical and efficient solution for oily wastewater treatment [31].

The separation principle of ultrafiltration is similar to that of microfiltration, primarily relying on physical sieving. However, ultrafiltration membranes have smaller pore sizes, making them particularly suitable for treating emulsified and dispersed oils. In oily wastewater treatment, ultrafiltration membranes can retain large molecular solutes ranging from 1 to 20 nm, including colloidal particles, bacteria, viruses, and proteins. This ensures a significant purification effect on water quality. Ultrafiltration membranes are typically asymmetric, consisting of a thin surface layer (less than 1 μm) and a porous support layer (approximately 125 μm). This structure provides efficient filtration performance and extends the membrane’s service life. In the treatment of oily wastewater, ultrafiltration membranes remove oils and effectively eliminate many suspended solids, bacteria, and organic pollutants, ensuring that the treated wastewater meets discharge standards or is suitable for further advanced treatment. Ultrafiltration membranes are usually prepared by phase inversion methods, with polysulfone and polyethersulfone being the most commonly used materials. These materials offer excellent chemical stability and mechanical strength, making them well-suited for various complex wastewater treatment environments [32].

#### 2.2.2. Classification Based on Membrane Material

Membrane materials and technologies for oil–water separation are chosen based on the unique properties of organic, inorganic, and composite membranes. The oil removal efficiencies and performance characteristics of these different membrane materials are summarized in Table 1, highlighting their strengths and suitability for various applications in oily wastewater treatment.

Polyester membrane materials are widely used in oily wastewater treatment due to their chemical stability, oil resistance, and mechanical strength, making them a preferred choice among organic membrane materials. Polyvinylidene fluoride (PVDF) is often utilized for treating high-concentration oily wastewater due to its exceptional chemical stability, strong oil resistance, and corrosion resistance. While polypropylene is a low-cost option with good corrosion resistance, its physical strength and chemical stability are comparatively lower. However, these properties can be enhanced to improve its suitability for oily wastewater treatment applications [43].

As shown in Table 1, the oil separation rate from oily wastewater using various composite membranes is generally higher than that achieved by organic or inorganic membranes made from a single material. This enhanced performance is due to the combined properties of the materials, which improve the membrane’s ability to effectively separate oil from water. Inorganic materials, such as alumina and silica ceramics, are commonly used in oily wastewater treatment due to their high interception efficiency for oil droplets and ability to effectively remove organic matter from water. Ceramic membranes, in particular, are known for their durability and resistance to harsh operating conditions. Carbon-based materials, such as graphene and carbon nanotubes, are also gaining attention for oily wastewater treatment due to their unique 2D structure and outstanding physical and chemical properties. Carbon membranes exhibit high porosity, a large specific surface area, and excellent hydrophobicity, enabling efficient separation of oil–water mixtures. Jafari et al. [44] developed tubular ceramic microfiltration membranes with an impressive oil rejection rate of 99.99%. This highlights the high efficiency of these membranes in treating oily wastewater and their potential for effective large-scale applications.

Composite membrane materials, which combine the advantages of polymer and ceramic membranes, offer enhanced fouling resistance, wear resistance, and chemical stability, making them suitable for treating various complex water qualities. For instance, the PVDF/TBC blend membranes demonstrated significantly higher water flux and superior fouling resistance than neat PVDF membranes. Notably, the blend membranes exhibited approximately 2.5 times higher water flux and ~99% oil rejection at 2 bar operating pressure when filtering an engine oil–water emulsion, outperforming the neat PVDF membranes [35]. These composite membranes typically comprise a specialized polymer matrix embedded with nanomaterials with specific separation functions. The preparation and design of composite membranes consider factors such as pore structure, chemical stability, and oil resistance. In treating oily wastewater, these membranes facilitate the coalescence and separation of oil droplets from the water, ensuring that the treated water meets discharge standards [43]. Wu et al. [45] developed diatomite/carbon composite microfiltration membranes as a novel and effective solution for oily wastewater purification. The results showed that these membranes achieved a maximum oil rejection of 98.9%, with a permeation flux of 147.86 kg/m^2^/h in a single-stage filtration process. This demonstrates the potential of MFCMs for efficient oily wastewater treatment.

#### 2.2.3. Other New Membrane Materials

In recent years, the application and preparation technology of superwetting materials in the field of oil–water separation has become a research hotspot. Superwetting materials of various sizes have shown great value and development potential, demonstrating outstanding performance in research and practical engineering applications. With the advancement of new technologies and interdisciplinary approaches, superwetting materials can efficiently separate various microemulsions in complex environments. Superwetting oil–water separation materials are primarily categorized into five types: superhydrophobic–superoleophilic materials, superhydrophilic–underwater superoleophobic materials, superhydrophobic–superoleophobic materials, “special” superwetting materials, and smart switchable superwetting materials [46].

Superhydrophobic–superoleophilic materials have been actively explored in recent years. For example, nickel particles with superhydrophobic–superoleophilic surfaces and multi-spiked nanostructures have been prepared using hydrothermal methods [47], and Janus particles have been synthesized without surfactants on their surface through ultrasonic atomization in a continuous flow process [48]. 2D materials achieve high separation fluxes and are classified into metal- and non-metal-based substrates. Metal-based superhydrophobic materials include bio-metal–organic framework (MOF) modified nickel (Ni@Bio-MOF) [49], while non-metal superhydrophobic materials include superhydrophobic ball clay-based ceramic hollow fiber membranes (HFM) developed by the Mohd Haiqal Abd Aziz team [50]. 3D materials, with their large specific surface area and high porosity, offer greater practicality. Examples include the photo-induced self-heating superhydrophobic sponge PDMS-MF [51] and superhydrophobic cotton aerogels prepared through a simple and mild dip-heating process [52].

The superhydrophilic–underwater superoleophobic materials are mainly divided into 2D and 3D types. Among them, 2D materials include the HAP-GO membrane prepared by introducing hydroxyapatite (HAP) and graphene oxide (GO) [53]. 3D superhydrophilic–underwater superoleophobic materials exhibit hydrophilicity in air and specific oil repellency underwater. There are three main types: organic, inorganic, and organic–inorganic hybrid materials, such as the graphene-based melamine sponge [54], underwater oleophobic–superhydrophilic Sr-MOF material [55], and renewable antibacterial nanofibrillated cellulose-based aerogel [56].

Superhydrophobic–superoleophobic materials exhibit dual repulsion towards both water and oil, with 3D materials possessing the advantages of low-cost raw materials, stable performance, and strong industrial applicability, such as air superamphiphobic 3D porous materials modified with Co_3_O_4_ nanoparticles [57].

“Special” superhydrophobic materials refer to some that exhibit three types of superhydrophobicity different from thermodynamic theories, such as underwater super-oil transportability and oil-phase superhydrophobicity [58]. Examples include the 2D amphiphilic superhydrophobic membranes of PVDF made by directly fixing nanoparticles through in situ polymerization and ionic crosslinking during the phase conversion process [59], as well as the underwater superhydrophobic 3D cellulose fiber prepared by the condensation reaction of melamine and formaldehyde [60]. 3D “special” superhydrophobic materials exhibit a stronger water absorption capacity.

Smart switchable superwetting materials change their wettability mechanisms in response to stimuli such as AgNPs-thiol modified PVDF electrospun nanofiber membranes with pH-responsive properties [61]. A TiO_2_ nanostructured coating mesh film allows the water permeability in oil to be intelligently controlled through UV irradiation and heating [62]. Smart responsive superwetting interface materials hold enormous potential and development space.

There are also dual-function membranes with surface wettability and photocatalytic self-cleaning capabilities [63], which use photocatalytic materials to achieve dual functions of oil-water separation and photocatalytic self-cleaning of organic pollutants accumulated on the membrane surface in oil-water systems. Wang et al. [64] prepared Co-Al-Ce LDHs on stainless steel nets using a hydrothermal synthesis method. The superwetting properties achieved on the membrane help separate light and heavy oil from water, with a retention rate higher than 99%. Additionally, under ultraviolet irradiation, over 98% of the organic methyl was successfully degraded within 2.5 h. In addition to the wettability of the oil–water separation membrane, to achieve cost effectiveness and recyclability, the addition of the photocatalytic self-cleaning function [65] may be a viable solution against scaling and harsh conditions.

### 2.3. Basic Properties of Membranes and Influencing Factors

The fundamental properties of membrane materials significantly influence their treatment effectiveness and efficiency. Key factors such as pore size, microstructure, surface roughness, flux, and surface charge collectively determine the filtration precision, fouling resistance, and stability of the membrane.

Pore size is a critical factor affecting membrane performance. The pore size dictates filtration precision, with oil droplets needing to be larger than the membrane pores for effective interception. Additionally, the pore size distribution considerably impacts the membrane’s fouling resistance. Membranes with a wide pore size distribution are more prone to clogging by particles, whereas those with a narrow pore size distribution can more effectively reduce fouling by repelling a broader range of particles. Research indicates that membranes with pore sizes greater than 80 nm are more susceptible to fouling, underscoring the importance of controlling pore size distribution to optimize fouling resistance [66].

The microstructure of the membrane directly influences its filtration precision and long-term stability. There is a close relationship between membrane porosity and surface roughness. As membrane porosity decreases, surface roughness increases, leading to higher transmembrane pressure and a greater likelihood of pollutant adsorption on the membrane surface. Therefore, membranes with high porosity and a narrow pore size distribution are generally preferred for treating oily wastewater. Surface roughness is another important factor that affects membrane performance [67]. Membranes with a rougher surface or stronger hydrophobicity are more susceptible to fouling because dirt can easily deposit on the membrane surface. While higher flux enhances wastewater treatment capacity, prioritizing high flux at the expense of filtration precision is not advisable. Thus, it is essential to comprehensively consider filtration precision, flux, and stability when selecting membrane materials [68].

Hydrophilicity is a key parameter used to characterize the surface properties of membranes. Higher hydrophilicity typically leads to a lower likelihood of membrane fouling and higher membrane flux [69]. Contact angle measurements are commonly used in studies to assess the surface hydrophilicity of membranes, providing insights into their performance and potential for scaling [70].

The surface charge of the membrane also plays a crucial role in membrane fouling. Membrane fouling is often facilitated by electrostatic attraction between the membrane and oil droplets. Studies have shown that membranes and oil droplets with similar charges can help prevent fouling by reducing this attraction [66].

### 2.4. Operating Modes of Membrane Separation Technology

Crossflow filtration is a key operating mode in membrane separation technology, where wastewater flows parallel to the membrane surface rather than perpendicularly through it. This flow pattern helps minimize the accumulation of contaminants on the membrane surface, thereby reducing the risk of membrane fouling and extending the membrane’s lifespan. Additionally, by adjusting operational parameters, such as applied pressure and wastewater flow rate, the separation performance of the membrane can be further optimized to achieve optimal oil–water separation efficiency. The crossflow filtration operating mode enhances treatment efficiency and ensures the stability and continuity of the membrane separation process, making it an effective method for treating oily wastewater [71]. Several studies have demonstrated that in the process of membrane crossflow filtration of oil and water, the behavior of oil droplets on the membrane surface can be used as a theoretical basis to predict and mitigate fouling. By deriving relevant physical formulas, these studies have identified the critical inlet pressure and crossflow velocity needed to dislodge fixed oil droplets, thereby minimizing membrane scaling issues as much as possible [72,73,74,75].

## 3. Current Research Status of Membrane Technology in Treating Oily Wastewater

### 3.1. Treatment of Dispersed Oil

Dispersed oil refers to small oil droplets or thin films of petroleum or its derivatives that form in water after discharge and can only coalesce through external forces, such as centrifugation [76]. When treating this type of dispersed oil, microfiltration membranes with pore sizes ranging from 10 to 100 μm are typically employed [77]. These microfiltration membranes effectively intercept and remove oil droplets from the water, thereby achieving water purification. In practical applications, microfiltration membranes are often used as a pretreatment step to reduce the load on subsequent treatment processes. In addition to microfiltration membranes, gravity sedimentation, coalescence, and air flotation are also cost-effective and practical technologies for treating dispersed oil. For instance, Basumatary et al. utilized mineral materials to manufacture tubular microfiltration membranes with smooth surfaces. When treating oily wastewater at three different concentrations, these membranes could completely remove the oil across all concentrations [78]. The membranes developed using this formulation exhibited a porosity of 59.27 ± 1.002% and a mechanical strength of 14.708 ± 2.603 MPa. The droplet size distribution of oil-in-water emulsions, measured using a laser particle size analyzer, indicated that the average droplet size ranged from 2.884 to 3.802 μm, significantly larger than the average pore size of the membrane. Based on the excellent oil removal efficiency demonstrated, it can be concluded that this microfiltration membrane holds industrial potential for treating oily wastewater.

### 3.2. Treatment of Emulsified Oil

Emulsified oil refers to a stable emulsion where oil droplets are surrounded by water, forming tiny oil droplets suspended in the water, making separation difficult. Unlike pure oil films or dispersed oil, emulsified oil does not readily separate or settle. In practical applications, Li et al. [79] developed a degradable regenerated lignocellulose composite membrane with a three-layer structure using an in situ regeneration method. This membrane exhibited excellent underwater superoleophobic performance, achieving high-efficiency separation of various emulsified oils and oil–water mixtures, with a separation efficiency exceeding 99.90%. Additionally, the oxygen-rich functional groups (such as hydroxyl, epoxy, and carboxyl groups) on the surface of graphene oxide impart hydrophilicity and strong interactions with various pollutants, facilitating their removal from wastewater [80]. Graphene and its derivatives have also been integrated into membrane separation processes, such as ultrafiltration and nanofiltration, to enhance filtration efficiency, selectivity, and antifouling performance. For example, Cheng et al. [81] designed a novel biodegradable nanofiber membrane (H-PLA-AS) with an asymmetric porous structure, featuring a large pore support layer and a small pore selective layer, using electrospinning technology. The H-PLA-AS membrane increased permeability by at least 25% and achieved a relative separation efficiency of approximately 99.6%, effectively removing oil droplets smaller than 150 nm. This membrane demonstrated stable separation performance, excellent antifouling properties, and the ability to degrade into lactic acid by proteases, allowing for reuse by biological organisms. The electrospinning process offers a promising new approach for developing separation membranes for oily wastewater treatment. Furthermore, Xie et al. [82] proposed a modified polyvinylidene fluoride (PVDF) membrane created through a simple inkjet printing process. This modified membrane exhibits hydrophilicity and superoleophobicity under gravity for various oil/water emulsions, with excellent water permeability and separation efficiency (>98.8%). Even after eight filtration cycles, the flux recovery rate remains high (>90.4%) while maintaining the original excellent separation efficiency. This modification technique can be applied to various substrates, addressing the demand for simple, low-cost, and rapid preparation of highly hydrophilic antifouling membranes for oil–water emulsion separation.

### 3.3. Treatment of Dissolved Oil

Dissolved oil refers tiny oil droplets that mix with water, which is difficult to separate. These droplets can remain suspended in water for extended periods, making them challenging to remove using conventional methods. Compared to emulsified oil, dissolved oil generally has lower toxicity because the smaller droplets have a larger surface area in contact with water, reducing the likelihood of ingestion by organisms. Recent research has focused on modifying membrane materials to enhance membrane flux and efficiency when treating oily wastewater [83]. Boyraz et al. [84] investigated the filtration performance of hybrid polyacrylonitrile nanofiber membranes for oily wastewater treatment. They found that the water permeability of these membranes increased by 2–8 times after alkaline treatment, while TiO_2_-grafted membranes exhibited a 2–13-fold increase in water permeability, making them promising materials for treating dissolved oil. Additionally, Earwood et al. [85] explored the surface modification of basalt fabric with TiO_2_ nanoparticles to create an efficient and low-cost 3D photothermal evaporator for oily wastewater treatment. Yadav et al. [86] used glutaraldehyde (GA)-modified electrospun polyvinyl alcohol (PVA) membranes to treat oil emulsions and recover fossil fuels. These modified membranes demonstrated superoleophobicity, effectively separating various oils (such as sesame oil, motor oil, mustard oil, and sunflower oil) from their emulsions, with a separation efficiency exceeding 99% for motor oil emulsions. Wang et al. [87] prepared a calcium alginate-based aerogel membrane (PANI@CA membrane) using aniline and sodium alginate as raw materials. This membrane could separate oil–water emulsions under gravity alone, achieving a separation efficiency of up to 99%. Moreover, the PANI@CA membrane effectively retained or adsorbed organic dyes and heavy metal ions, offering advantages such as low cost, simple preparation, good stability, and high recyclability.

## 4. Combined Membrane Technology Processes for Treating Oily Wastewater

### 4.1. Pretreatment Process

Due to the presence of poorly soluble organic substances, high concentrations of ammonia nitrogen, suspended solids, oils, and small amounts of toxic pollutants in oily wastewater, pretreatment is essential before applying subsequent treatment processes. This approach enhances treatment efficiency and improves the quality of the effluent. For instance, Sulei et al. [88] implemented a pretreatment double-membrane process for advanced wastewater treatment at a refinery. The pretreatment technology combined physical and biochemical methods, including coagulation air flotation tanks, aerated biological filters, precision filters, and multi-chamber biological activated carbon filters, to preliminarily treat chemical refinery wastewater. This pretreatment ensured that the water quality met the requirements for the double membrane technology, thereby reducing its operational load. Over several years of operation, the effluent quality remained consistently high, with pollutant removal rates exceeding 92%. Similarly, Hu et al. conducted a wastewater treatment project at an oil processing company in Henan, where the wastewater exhibited high oil and COD content [89]. The pretreatment design included a two-stage oil separation tank, an air flotation tank with both induced air and dissolved air, and the addition of ceramic membrane filters to remove residual oils and scum from the wastewater. They also employed a steam stripping tower for purification, followed by a sedimentation tank and a secondary air flotation tank to reduce suspended solids content. The treated effluent achieved oil and COD removal rates of 99.5% and 99.6%, respectively, demonstrating significant treatment effectiveness. However, a small amount of anionic polyacrylamide and crude oil (the main organic matter on the membrane surface) can still pass through the ultrafiltration barrier, resulting in severe contamination of the nanofiltration membrane as well as polyvalent elements and silica. However, no pretreatment methods are specifically designed to remove Al, Fe, and Si. Therefore, it is critical to optimize existing pretreatment strategies or develop cost-effective procedures to guarantee the removal of Al, Fe, and Si from feedstock at NF facilities [90].

### 4.2. Membrane Bioreactor

Membrane Bioreactors (MBRs) combine traditional membrane separation technology with biological treatment processes. In an MBR, microorganisms in the bioreactor degrade oil substances, converting them into water-soluble organic matter and other biomass. The treated wastewater is then passed through membrane modules for physical or chemical separation, effectively removing oils. This technology effectively eliminates suspended solids and dissolved organic matter from the water while retaining most bacteria and viruses and, thus, achieved higher water quality standards. However, membrane fouling can easily occur during operation, necessitating further optimization [91]. At the same time, membrane contamination and high operating costs remain the biggest challenges.

Safdari et al. developed a side-stream MBR process using blended polyetherimide-sulfonated polyetheretherketone (PEI-sPEEK) hollow fiber membranes for treating oily wastewater [92]. This modified membrane featured a large finger-like open structure with a total porosity of approximately 80% and an average surface pore size of 10 nm. In the MBR process, the improved membrane achieved oil removal rates, COD reduction, TSS reduction, and turbidity reduction rates of approximately 99.9%, 99.7%, 98.9%, and 99.7%, respectively. The membrane also demonstrated good antifouling performance, with a flux recovery rate (FRR) of 80.2%, where cake layer filtration was identified as the primary fouling mechanism. Due to its enhanced performance, this modified membrane offers new possibilities for efficiently treating industrial oily wastewater. Bhattacharyya et al. compared the treatment efficiency and capacity of a submerged hollow fiber (HF) membrane filtration system with an MBR system [93]. The MBR system outperformed the membrane filtration system in removing total petroleum hydrocarbons and polycyclic aromatic hydrocarbons (PAHs). The MBR system showed significant potential, particularly when the initial oil concentration was below 30 ppm. The pilot-scale membrane filtration system effectively removed most PAHs from the wastewater to levels acceptable for Canadian surface waters.

### 4.3. Photocatalytic Membrane Reactor

A Photocatalytic Membrane Reactor (PMR) is an advanced wastewater treatment process that combines photocatalytic and membrane separation technologies. In this process, membrane separation technology first removes oil droplets and suspended solid particles from the water. Then, photocatalytic technology generates highly reactive hydroxyl radicals (·OH), which decompose organic pollutants in the water into harmless substances. During the photocatalytic reaction, the membrane in the reactor continues to function as a separation medium, retaining small organic molecules generated during decomposition and intercepting bubbles and particulates formed in the photocatalytic reaction, thereby improving effluent quality.

The advantages of PMR include energy savings, a reduced installation footprint, and the avoidance of additional costs associated with sedimentation, coagulation, and flocculation [94]. Photocatalysts in PMR systems can absorb energy from sunlight, visible light, and ultraviolet light, and various polymer and inorganic membranes exhibit excellent self-cleaning and antifouling capabilities under UV and visible light irradiation. It is important to note that the light source, configuration, and design of photocatalysts are important for the effective degradation of target contaminants in oily wastewater. The degree of mineralization of contaminants and the type and toxicity of by-products resulting from incomplete degradation are key issues to be faced in implementing every PMR system.

For example, Moslehyani et al. [95] developed a polyvinylidene fluoride/multi-walled carbon nanotube (PVDF/MWCNT) nanocomposite photocatalytic membrane system for treating refinery wastewater. This system effectively retained TiO_2_, with a removal rate exceeding 99%. The photocatalytic reaction degraded more than 90% of pollutants, and the ultrafiltration permeation tank almost completely removed the contaminants, demonstrating excellent antifouling performance. Alias et al. [96] prepared a photocatalytic nanofiber by exfoliating graphitic carbon nitride into nanosheets and incorporating them into polyacrylonitrile nanofibers for treating oily wastewater. This nanofiber achieved an 85.4% degradation rate of oily wastewater under visible light irradiation, and a 96.6% degradation rate under UV light. The high photocatalytic degradation ability was attributed to the oil adsorption capacity of polyacrylonitrile nanofibers and the oil photodegradation capacity of graphitic carbon nitride. The synergistic effect of these properties resulted in the effective degradation of oily wastewater by the photocatalytic nanofibers. Zangeneh et al. developed a photocatalytic self-cleaning polyethersulfone (PES) nanofiltration membrane doped with triple metal-non-metal-doped TiO_2_ (K-B-N-TiO_2_) to treat oily wastewater from palm oil mills [97]. The study found that the addition of K-B-N-TiO_2_ nanoparticles (0.5 wt%) improved the water flux of the modified membrane due to their hydrophilicity and the photocatalytic activity of the photocatalyst. The maximum permeate flux of the treated wastewater was 27 kg/m^2^·h, with a dye removal rate of 98% and a COD removal rate of 90%.

### 4.4. Combined Treatment Processes for Industrial Oily Wastewater

The treatment process for industrial oily wastewater typically involves a combination of multiple methods to address the complex composition and varying particle sizes of the contaminants. Initially, large particles are removed through sedimentation, followed by biological or chemical treatments to further eliminate smaller pollutants. Membrane separation is often used as the final step to filter out the smallest, most difficult-to-treat particles.

For example, Zhao et al. employed a treatment process involving a “regulating tank + air flotation + A/O + MBR” configuration [98]. The process begins with an impeller air flotation (IAF) device, which is a specialized suspended solids separation and purification system designed to remove solids, colloids, and oils from wastewater. This is followed by hydrolytic acidification in an anoxic stage (Tank A) and biological contact oxidation in an aerobic stage (Tank O). These stages utilize facultative and aerobic bacteria to thoroughly decompose biodegradable pollutants in the water. The membrane bioreactor (MBR) replaces the traditional sedimentation tank with a hollow fiber membrane, enabling efficient solid–liquid separation and ensuring that the effluent meets discharge standards. In practice, the wastewater first passes through an oil separator to remove large suspended oil particles and then flows through a screen to eliminate large debris. After that, it enters a regulating tank for water quality adjustment and flow equalization before being pumped into the air flotation unit, which primarily removes oils and suspended solids. The wastewater then undergoes anaerobic treatment in Tank A and aerobic treatment in Tank O before entering the MBR tank. The treated water is finally stored in a clear water tank and discharged in compliance with environmental standards. The operational results demonstrated high removal rates for COD, SS, BOD_5_, NH_3_-N, and animal and vegetable oils, achieving 95.8%, 97.9%, 97.4%, 76.5%, and 96.8%, respectively.

For chemical oily wastewater, which typically contains suspended solids and oils with poor biodegradability, Li et al. utilized a combination of oil separation and coagulation processes. The wastewater is initially pumped into a secondary sedimentation oil separation tank, where heavier particles such as sand settle quickly, and part of the oil is removed. The effluent then enters a coagulation–flocculation tank, where organic and inorganic substances combine to form flocs that are subsequently separated from the water, removing most of the COD. The treated wastewater is then passed through a multi-media filter filled with quartz sand and anthracite to remove any remaining particulates and flocs, resulting in a clear effluent [99].

Refinery wastewater, a complex mixture of hydrocarbons, sulfides, ammonia, oils, suspended and dissolved solids, and heavy metals, presents significant treatment challenges due to the toxic and recalcitrant nature of these pollutants. Refineries typically produce wastewater at approximately 0.4–1.5 times the volume of refined oil [100]. To address this, Bai et al. developed a “pretreatment unit upgrade + biological treatment unit upgrade” solution [101]. The pretreatment unit employs a “tank-in-tank + micro-nano air flotation” process, while the biological oxidation unit utilizes an “MBBR tank + automatic dosing system for ammonia nitrogen removal.” The tank-in-tank + micro-nano air flotation enhances oil separation in the pretreatment unit and improves the wastewater treatment plant’s resilience to shock loads. The Moving Bed Biofilm Reactor (MBBR) combines biofilm and activated sludge processes within the same unit, increasing the treatment capacity and effectiveness of the biological treatment. After treatment with this system, the wastewater typically achieves a COD value of less than 30 mg/L and an ammonia nitrogen content of less than 2.5 mg/L, meeting stringent discharge requirements.

## 5. Challenges and Prospects of Membrane Technology in Treating Oily Wastewater

In the treatment of oily wastewater, membrane technology is widely applied due to its high efficiency and precision. However, the technology still faces significant challenges, such as reduced flux, increased energy consumption, and scaling caused by the accumulation of oil droplets on the membrane surface. These issues lead to decreased membrane lifespan and efficiency and contribute to a range of problems related to membrane fouling [90]. In certain industrial scenarios, such as power transmission and transformation projects, the complexity of oily wastewater—where oily substances are mixed with other pollutants—further increases the risk of membrane fouling and clogging, thereby shortening membrane lifespan and raising maintenance costs. Additionally, oils and other chemicals in the wastewater can easily adhere to the membrane surface, exacerbating membrane fouling and significantly impacting treatment efficiency and effectiveness [102]. Echakouri et al. [103] introduced a new physical method for controlling membrane fouling, called the periodic transmembrane pressure technique (PTMP). In this method, membrane cleaning occurs periodically when the transmembrane pressure (TMP) drops to zero. The study compared PTMP’s performance with backwashing and pulsating flow techniques. The results indicate that PTMP achieves higher total osmotic volume, steady-state osmotic flux, residual flux, and dirt reversibility than the other two methods. Another method, periodic feed pressure technology (PFPT), mitigates membrane fouling by periodically setting the TMP to zero, allowing stationary oil droplets to detach from the membrane surface. PFPT does not damage the supporting layer of the polymer membrane and results in a much cleaner membrane surface compared to conventional filtration processes. Additionally, it eliminates the need for extra chemicals or equipment, making it a promising solution for membrane fouling [104].

Moreover, the challenging site environments and the variable volume and composition of wastewater make treatment more difficult. Site conditions often restrict the use of large treatment equipment, necessitating more flexible and convenient membrane treatment devices, which adds to the technological challenges. Therefore, applying membrane technology to treat oily wastewater in large-scale projects requires optimization and improvement to address these specific challenges.

Modifying membrane materials through surface and chemical modifications can mitigate membrane scaling during the treatment of industrial oily wastewater, thereby enhancing membrane performance. These modifications can give membranes better permeability, biofouling resistance, and hydrophilicity. Additionally, research can explore using agricultural by-products as adsorbents to achieve low-cost, sustainable, and environmentally friendly treatment of industrial oily wastewater [105]. Many current methods are only suitable for specific types of oily particles, necessitating the combination of various methods to treat more complex mixtures. Integrating traditional treatment methods with modern technologies can enhance oil removal performance. Sustainable, biocompatible, and biodegradable methods should be explored for oily wastewater treatment, particularly those involving the combination of microorganisms and membranes, which promise to reduce energy costs, improve treatment efficiency, and minimize waste generation while reducing pollutant emissions [106]. Furthermore, the integration of intelligent control systems with advanced membrane technology can enable real-time monitoring and optimization of the membrane separation process for industrial oily wastewater, thereby improving efficiency and stability while reducing pollution and operational costs. This advancement drives the wastewater membrane separation industry toward greater intelligence and efficiency.

Additionally, to mitigate both oil and water pollution at their sources, it is imperative to minimize water usage within the oil industry. The strategies for reducing water consumption in the petroleum sector are primarily encompassed in two broad categories: production process optimization and enhancing legislative and regulatory frameworks. Initially, concerning production process optimization, water consumption can be decreased through the refinement of operational procedures, the implementation of water recycling systems, and the modernization of equipment. Regarding the enhancement of laws and regulations, it is essential for the government to develop comprehensive and long-term water resource management strategies. Furthermore, industrial entities must establish robust and compliant water monitoring systems to facilitate real-time tracking and analysis of water usage throughout the production cycle, thereby enabling the prompt identification and resolution of water wastage issues [107,108].

## 6. Conclusions

Membrane separation technology is widely utilized in oily wastewater treatment due to its broad applicability, effective treatment outcomes, and ease of operation. Various treatment methods for different sources and characteristics of oily wastewater are discussed, and the characteristics of different membrane separation technologies and their application are summarized in this review. Despite its many advantages, practical applications must address and optimize several challenges, including membrane fouling, aging, and high energy consumption. The future development of membrane treatment technology for oily wastewater should focus on practical applications, optimizing the integration of various technologies, and identifying new and efficient wastewater treatment solutions that require minimal investment.

## Figures and Tables

**Table 1:** Merits and demerits of various membrane separation technologies for oily wastewater treatment.

Membrane Material		WCA (Degree)	Flux (Lm^−2^ h^−1^)	Rejection of Oil (%)	Durability Expressed as the Cycle Numbers	Fouling Resistance	References
Type	Membrane						
Organic membranes	PAN	151	26,160	99.9	/	/	[33]
	isotropic PES	39	4721	99	/	irreversible fouling	[34]
	anisotropic PES	70	347	99 (at low oil concentrations)	/	irreversible fouling	
	Neat PVDF	80	130	94.4	pore blocking after 60 min	poor fouling resistance	[35]
Inorganic membranes	Al_2_O_3_	23.4	139.4	90.7	59.1% decreased after 150 min	irreversible adsorption fouling	[36]
	SiC	11.3	163.9	93.5	36.7% decreased after 150 min	/	
	Ceramic hollow fiber	-	182	97.1	/	/	[37]
Composite membranes	PDA@PLA NF	26	990	99.5	/	easily recovered	[38]
	β-CD-PDA@PLA	0	1500	99.5	30	easily recovered	[38]
	PVDF/TBC blends	71~75	280~360	97.4~99.5	pore blocking after 60 min	poor fouling resistance	[35]
	PES/SPES/MPN	63.3	>1500	>99.5	flux recovery over 82% after 5 cycles	/	[39]
	TiO_2_/SiO_2_	0	3636	91	/	/	[40]
	Anthurium andraeanum-like Fe_3_O_4_-attapulgite fibers	0	209	98.7	/	easily recovered by 86.5%	[41]
	CC@TiO_2_-Cu	0	8~10	99.3%	rejection of oil reached 99.6% after 80 cycles	excellent photocatalytic and self-cleaning performances under visible light driven	[42]

Note: PAN, polyacrylonitrile; PES, polyethersulfone; PVDF, poly (vinylidene fluoride); PDA@PLA NF membranes, PLA nanofiber membrane with dopamine; β-CD, β-cyclodextrins;TBC, tri-block copolymer; PES, polyether sulfone; SPES, sulfonated polyether sulfone; MPN, metal-polyphenol networks.

## Data Availability

The original data presented in the study are included in the article.
